# The *LRRK2* p.L1795F variant causes Parkinson’s disease in the European population

**DOI:** 10.1038/s41531-025-00896-2

**Published:** 2025-03-25

**Authors:** Lara M. Lange, Kristin Levine, Susan H. Fox, Connie Marras, Nazish Ahmed, Nicole Kuznetsov, Dan Vitale, Hirotaka Iwaki, Katja Lohmann, Luca Marsili, Alberto J. Espay, Peter Bauer, Christian Beetz, Jessica Martin, Stewart A. Factor, Lenora A. Higginbotham, Honglei Chen, Hampton Leonard, Mike A. Nalls, Niccolo E. Mencacci, Huw R. Morris, Andrew B. Singleton, Christine Klein, Cornelis Blauwendraat, Zih-Hua Fang, the Global Parkinson’s Genetics Program (GP2), Lara M. Lange, Kristin Levine, Dan Vitale, Hirotaka Iwaki, Katja Lohmann, Luca Marsili, Alberto J. Espay, Honglei Chen, Hampton Leonard, Mike A. Nalls, Niccolo E. Mencacci, Huw R. Morris, Andrew B. Singleton, Christine Klein, Cornelis Blauwendraat, Zih-Hua Fang, Emilia M. Gatto, Marcelo Kauffman, Samson Khachatryan, Zaruhi Tavadyan, Claire E. Shepherd, Julie Hunter, Kishore Kumar, Melina Ellis, Miguel E. Rentería, Sulev Koks, Alexander Zimprich, Artur F. Schumacher-Schuh, Carlos Rieder, Paula Saffie Awad, Vitor Tumas, Sarah Camargos, Edward A. Fon, Oury Monchi, Ted Fon, Benjamin Pizarro Galleguillos, Patricio Olguin, Marcelo Miranda, Maria Leonor Bustamante, Pedro Chana, Beisha Tang, Huifang Shang, Jifeng Guo, Piu Chan, Wei Luo, Gonzalo Arboleda, Jorge Orozco, Marlene Jimenez del Rio, Alvaro Hernandez, Mohamed Salama, Walaa A. Kamel, Yared Z. Zewde, Alexis Brice, Jean-Christophe Corvol, Ana Westenberger, Eva-Juliane Vollstedt, Harutyun Madoev, Joanne Trinh, Johanna Junker, Anastasia Illarionova, Brit Mollenhauer, Franziska Hopfner, Günter Höglinger, Manu Sharma, Thomas Gasser, Sergiu Groppa, Albert Akpalu, Georgia Xiromerisiou, Georgios Hadjigorgiou, Efthymios Dadiotis, Ioannis Dagklis, Ioannis Tarnanas, Leonidas Stefanis, Maria Stamelou, Alex Medina, Germaine Hiu-Fai Chan, Nelson Yuk-Fai Cheung, Nancy Ip, Phillip Chan, Xiaopu Zhou, Asha Kishore, Divya KP, Pramod Pal, Prashanth Lingappa Kukkle, Roopa Rajan, Rupam Borgohain, Mehri Salari, Andrea Quattrone, Monica Gagliardi, Enza Maria Valente, Micol Avenali, Grazia Annesi, Lucilla Parnetti, Tommaso Schirinzi, Manabu Funayama, Nobutaka Hattori, Tomotaka Shiraishi, Altynay Karimova, Gulnaz Kaishibayeva, Cholpon Shambetova, Rejko Krüger, Ai Huey Tan, Azlina Ahmad-Annuar, Shen-Yang Lim, Yi Wen Tay, Mohamed Ibrahim Norlinah, Nor Azian Abdul Murad, Shahrul Azmin, Wael Mohamed, Daniel Martinez-Ramirez, Mayela Rodriguez-Violante, Paula Reyes-Pérez, Bayasgalan Tserensodnom, Rajeev Ojha, Tim J. Anderson, Toni L. Pitcher, Arinola Sanyaolu, Njideka Okubadejo, Oluwadamilola Ojo, Jan O. Aasly, Lasse Pihlstrøm, Manuela Tan, Shoaib Ur-Rehman, Mario Cornejo-Olivas, Maria Leila Doquenia, Raymond Rosales, Angel Vinuela, Elena Iakovenko, Bashayer Al Mubarak, Muhammad Umair, Eng-King Tan, Jia Nee Foo, Ferzana Amod, Jonathan Carr, Soraya Bardien, Beomseok Jeon, Yun Joong Kim, Esther Cubo, Ignacio Alvarez, Janet Hoenicka, Katrin Beyer, Maria Teresa Periñan, Pau Pastor, Sarah El-Sadig, Kajsa Brolin, Christiane Zweier, Paul Krack, Gerd Tinkhauser, Chin-Hsien Lin, Pin-Jui Kung, Hsiu-Chuan Wu, Ruey-Meei Wu, Yihru Wu, Rim Amouri, Samia Ben Sassi, A. Nazlı Başak, Özgür Öztop Çakmak, Sibel Ertan, Gencer Genc, Alastair Noyce, Sumit Dey, Alejandro Martínez-Carrasco, Anette Schrag, Anthony Schapira, Eleanor J. Stafford, Henry Houlden, John Hardy, Kin Ying Mok, Mie Rizig, Nicholas Wood, Olaitan Okunoye, Rauan Kaiyrzhanov, Rimona Weil, Simona Jasaityte, Vida Obese, Camille Carroll, Claire Bale, Donald Grosset, Nigel Williams, Patrick Alfryn Lewis, Seth Love, Simon Stott, Caroline B. Pantazis, Kate Andersh, Laurel Screven, Sara Bandres-Ciga, Ignacio Juan Keller Sarmiento, Alyssa O’Grady, Bernadette Siddiqi, Bradford Casey, Brian Fiske, Charisse Comart, Justin C. Solle, Kaileigh Murphy, Maggie Kuhl, Naomi Louie, Sohini Chowdhury, Todd Sherer, Andrew K. Sobering, Cabell Jonas, Carlos Cruchaga, Laura Ibanez, Claire Wegel, Tatiana Foroud, Deborah Hall, Dena Hernandez, Jonggeol Jeff Kim, Yeajin Song, Ejaz Shiamim, Ekemini Riley, Geidy E. Serrano, Ignacio F. Mata, Miguel Inca-Martinez, Jared Williamson, Joseph Jankovic, Joshua Shulman, Kamalini Ghosh Galvelis, Karen Nuytemans, Karl Kieburtz, Katerina Markopoulou, Kenneth Marek, Lana M. Chahine, Lauren Ruffrage, Lisa Shulman, Marissa Dean, Matthew Farrer, Megan J. Puckelwartz, Steven Lubbe, Roger Albin, Roy Alcalay, Ruth Walker, Sonya Dumanis, Tao Xie, Thomas Beach, Faraz Faghri, Mary B. Makarious, Mathew Koretsky, Duan Nguyen, Toan Nguyen, Masharip Atadzhanov

**Affiliations:** 1 Institute of Neurogenetics, University of Luebeck, Luebeck, Germany; 2 Department of Neurology, University Hospital Schleswig-Holstein, Luebeck, Germany; 3 Laboratory of Neurogenetics, National Institute on Aging, National Institutes of Health, Bethesda, MD USA; 4 DataTecnica, Washington, DC USA; 5 Center for Alzheimer’s and Related Dementias (CARD), National Institute on Aging and National Institute of Neurological Disorders and Stroke, National Institutes of Health, Bethesda, MD USA; 6 Edmond J. Safra Program in Parkinson’s Disease and the Morton and Gloria Shulman Movement Disorders Clinic, Toronto Western Hospital, University Health Network, University of Toronto, Toronto, ON Canada; 7 University of Cincinnati, Cincinnati, OH USA; 8 CENTOGENE GmbH, Rostock, Germany; 9 Department of Neurology, Emory University School of Medicine, Atlanta, GA USA; 10 Department of Epidemiology and Biostatistics, Michigan State University, Michigan, MI USA; 11 Department of Neurology, Northwestern University Feinberg School of Medicine, Chicago, IL USA; 12 Department of Clinical and Movement Neurosciences, UCL Queen Square Institute of Neurology, London, UK; 13 UCL Movement Disorders Centre, University College London, London, UK; 14 German Center for Neurodegenerative Diseases (DZNE), Tübingen, Germany; 15 Sanatorio de la Trinidad Mitre – INEBA, Buenos Aires, Argentina; 16 Hospital JM Ramos Mejia, Buenos Aires, Argentina; 17 Somnus Neurology Clinic, Yerevan, Armenia; 18 Neuroscience Research Australia, Sydney, NSW Australia; 19 ANZAC Research Institute, Concord, NSW Australia; 20 Garvan Institute of Medical Research and Concord Repatriation General Hospital, Darlinghurst, NSW Australia; 21 Concord Hospital, Concord, NSW Australia; 22 QIMR Berghofer Medical Research Institute, Herston, QLD Australia; 23 Murdoch University, Perth, Western Australia Australia; 24 Medical University Vienna, Vienna, Austria; 25 Universidade Federal do Rio Grande do Sul / Hospital de Clínicas de Porto Alegre, Porto Alegre, Brazil; 26 Federal University of Health Sciences of Porto Alegre, Porto Alegre, Brazil; 27 Universidade Federal do Rio Grande do Sul, Porto Alegre, Brazil; 28 University of São Paulo, São Paulo, Brazil; 29 Universidade Federal de Minas Gerais, Belo Horizonte, Brazil; 30 Montreal Neurological Institute, Montreal, Quebec Canada; 31 Institut universitaire de gériatrie de Montréal, Montreal, Quebec Canada; 32 McGill University, Montreal, Quebec Canada; 33 Universidad de Chile, Santiago, Chile; 34 Fundación Diagnosis, Santiago, Chile; 35 Faculty of Medicine Universidad de Chile, Santiago, Chile; 36 CETRAM, Santiago, Chile; 37 Central South University, Changsha, China; 38 West China Hospital Sichuan University, Chengdu, China; 39 Xiangya Hospital, Changsha, China; 40 Capital Medical University, Beijing, China; 41 Zhejiang University, Hangzhou, China; 42 Universidad Nacional de Colombia, Bogotá, Colombia; 43 Fundación Valle del Lili, Santiago De Cali, Colombia; 44 University of Antioquia, Medellin, Colombia; 45 University of Costa Rica, San Jose, Costa Rica; 46 The American University in Cairo, Cairo, Egypt; 47 Beni-Suef University, Beni Suef, Egypt; 48 Addis Ababa University, Addis Ababa, Ethiopia; 49 Paris Brain Institute, Paris, France; 50 Sorbonne Université, Paris, France; 51 University Medical Center Göttingen, Göttingen, Germany; 52 Department of Neurology, University Hospital, LMU Munich, Munich, Germany; 53 University of Tübingen, Tübingen, Germany; 54 University of Mainz, Mainz, Germany; 55 University of Ghana Medical School, Accra, Ghana; 56 University of Thessaly, Volos, Greece; 57 Aristotle University of Thessaloniki, Thessaloniki, Greece; 58 Ionian University, Corfu, Greece; 59 Biomedical research Foundation of the Academy of Athens, Athens, Greece; 60 Diagnostic and Therapeutic Centre HYGEIA Hospital, Marousi, Greece; 61 Hospital San Felipe, Tegucigalpa, Honduras; 62 Queen Elizabeth Hospital, Kowloon, Hong Kong; 63 The Hong Kong University of Science and Technology, Kowloon, Hong Kong; 64 Aster Medcity, Kochi, India; 65 Sree Chitra Tirunal Institute for Medical Sciences and Technology, Thiruvananthapuram, India; 66 National Institute of Mental Health & Neurosciences, Bengaluru, India; 67 Manipal Hospital, Delhi, India; 68 All India Institute of Medical Sciences, Delhi, India; 69 Nizam’s Institute Of Medical Sciences, Hyderabad, India; 70 Shahid Beheshti University of Medical Science, Tehran, Iran; 71 Magna Graecia University of Catanzaro, Catanzaro, Italy; 72 University of Pavia, Pavia, Italy; 73 National Research Council, Cosenza, Italy; 74 University of Perugia, Perugia, Italy; 75 University of Rome Tor Vergata, Rome, Italy; 76 Juntendo University, Tokyo, Japan; 77 Jikei University School of Medicine, Tokyo, Japan; 78 Institute of Neurology and Neurorehabilitation, Almaty, Kazakhstan; 79 Kyrgyz State Medical Academy, Bishkek, Kyrgyzstan; 80 University of Luxembourg, Luxembourg, Luxembourg; 81 University of Malaya, Kuala Lumpur, Malaysia; 82 Universiti Kebangsaan Malaysia, Selangor, Malaysia; 83 UKM Medical Molecular Biology Institute, Kuala Lumpur, Malaysia; 84 Universiti Kebangsaan Malaysia Medical Centre, Kuala Lumpur, Malaysia; 85 International Islamic University, Kuala Lumpur, Malaysia; 86 Tecnologico de Monterrey, Monterrey, Mexico; 87 Instituto Nacional de Neurologia y Neurocirugia, Mexico City, Mexico; 88 Universidad Nacional Autónoma de México, Mexico City, Mexico; 89 Mongolian National University of Medical Sciences, Ulaanbaatar, Mongolia; 90 Tribhuvan University, Kirtipur, Nepal; 91 University of Otago, Dunedin, New Zealand; 92 University of Lagos, Lagos, Nigeria; 93 Norwegian University of Science and Technology, Trondheim, Norway; 94 Oslo University Hospital, Oslo, Norway; 95 University of Science and Technology Bannu, Bannu, Pakistan; 96 Universidad Cientifica del Sur, Lima, Peru; 97 Metropolitan Medical Center, Manila, Philippines; 98 University of Puerto Rico, San Juan, Puerto Rico; 99 Research Center of Neurology, Moscow, Russia; 100 King Faisal Specialist Hospital and Research Center, Riyadh, Saudi Arabia; 101 King Abdullah International Medical Research Center, Jeddah, Saudi Arabia; 102 National Neuroscience Institute, Singapore, Singapore; 103 Nanyang Technological University, Singapore, Singapore; 104 University of KwaZulu-Natal, Durban, South Africa; 105 Stellenbosch University, Stellenbosch, South Africa; 106 Seoul National University Hospital, Seoul, South Korea; 107 Yongin Severance Hospital, Seoul, South Korea; 108 Hospital Universitario Burgos, Burgos, Spain; 109 University Hospital Mutua Terrassa, Barcelona, Spain; 110 Institut de Recerca Sant Joan de Deu, Barcelona, Spain; 111 Research Institute Germans Trias i Pujol, Barcelona, Spain; 112 Instituto de Biomedicina de Sevilla, Seville, Spain; 113 University Hospital Germans Trias i Pujol, Barcelona, Spain; 114 Faculty of medicine university of Khartoum, Khartoum, Sudan; 115 Lund University, Lund, Sweden; 116 Inselspital Bern, University of Bern, Bern, Switzerland; 117 University Hospital Bern, Bern, Switzerland; 118 National Taiwan University Hospital, Taipei City, Taiwan; 119 National Taiwan University, Taipei City, Taiwan; 120 Chang Gung Memorial Hospital, Taoyuan City, Taiwan; 121 National Institute Mongi Ben Hamida of Neurology, Tunis, Tunisia; 122 Koç University, Istanbul, Turkey; 123 Şişli Etfal Training and Research Hospital, Istanbul, Turkey; 124 Queen Mary University of London, London, UK; 125 University College London, London, UK; 126 University of Plymouth, Plymouth, UK; 127 Parkinson’s UK, London, UK; 128 University of Glasgow, Glasgow, UK; 129 Cardiff University, Cardiff, UK; 130 Royal Veterinary College University of London, London, UK; 131 University of Bristol, Bristol, UK; 132 Cure Parkinson’s, London, UK; 133 National Institutes of Health, Bethesda, MD USA; 134 The Michael J. Fox Foundation for Parkinson’s Research, New York, NY USA; 135 Augusta University / University of Georgia Medical Partnership, Augusta, GA USA; 136 Mid-Atlantic Permanente Medical Group, Bethesda, MD USA; 137 Washington University, St. Louis, MO USA; 138 Indiana University, Bloomington, IN USA; 139 Indiana University School of Medicine, Indianapolis, IN USA; 140 Rush University, Chicago, IL USA; 141 Kaiser Permanente, Oakland, CA USA; 142 Coalition for Aligning Science, Washington, WA USA; 143 Banner Sun Health Research Institute, Sun City, AZ USA; 144 Cleveland Clinic, Cleveland, OH USA; 145 Baylor College of Medicine, Houston, TX USA; 146 Parkinson’s Foundation, Princeton, NJ USA; 147 University of Miami Miller School of Medicine, Miami, FL USA; 148 Beth Israel Deaconess Medical Center, Boston, MA USA; 149 North Shore University Health System, Chicago, IL USA; 150 Institute for Neurodegenerative Disorders, New Haven, CT USA; 151 University of Pittsburgh, Pittsburgh, PA USA; 152 University of Alabama at Birmingham, Birmingham, AL USA; 153 University of Maryland, Baltimore, MD USA; 154 University of Florida – Neurology, Gainesville, FL USA; 155 Northwestern University, Chicago, IL USA; 156 University of Michigan, Ann Arbor, MI USA; 157 Columbia University, New York, NY USA; 158 James J. Peters Veterans Affairs Medical Center, New York, NY USA; 159 Aligning Science Across Parkinson’s, Washington, WA USA; 160 University of Chicago, Chicago, IL USA; 161 Sun Health Research Institution, Sun City, AZ USA; 162 Hue University, Huế, Vietnam; 163 University of Zambia, Lusaka, Zambia

**Keywords:** Mutation, Parkinson's disease

## Abstract

*LRRK2*-PD represents the most common form of autosomal dominant Parkinson’s disease. We identified the *LRRK2* p.L1795F variant in three families and six additional unrelated cases using genetic data from over 50,000 individuals. Carriers with available genotyping data shared a common haplotype. The clinical presentation resembles other *LRRK2*-PD forms. Combined with published functional evidence showing strongly enhanced LRRK2 kinase activity, we provide evidence that *LRRK2* p.L1795F is pathogenic.

Pathogenic variants in the *LRRK2* gene are among the most common causes of autosomal dominant Parkinson’s disease (PD)^1,2^ and are thought to act through a gain-of-function mechanism that increases kinase activity^3^. The *LRRK2* p.L1795F variant (chr12:40322386:G:T, hg38, rs111910483) has been shown to significantly enhance kinase activity, supporting its pathogenic role^4^. It was previously identified in eight PD cases from 2007 to 2019^5–7^, and most recently 2024^8^ as well as suggested as a genetic risk factor with an odds ratio (OR) of 2.5^9^. However, insufficient evidence of segregation precluded this variant from being considered “pathogenic”. Determining pathogenicity is crucial for diagnosis, genetic counseling, and even more for treatment, particularly now that *LRRK2*-specific clinical trials are underway^10,11^.

We screened a large cohort of PD cases and controls with short-read whole-genome sequencing (WGS) data, including 16,351 individuals from GP2 release 8 (DOI 10.5281/zenodo.13755496) and AMP-PD release 4 (for details see Methods and Supplementary Table 1) to identify recurrent rare coding variants of unknown significance co-segregating with PD in known PD genes (*LRRK2, SNCA, VPS35, PINK1, PRKN, PARK7*, and *GBA1*). We identified nine carriers of the *LRRK2* p.L1795F variant (ENST00000298910.12:c.5385 G > T; chr12:40322386:G:T; Supplementary Figs. 1-6). Of these carriers, we identified two families based on kinship inference using genetic data (Fig. 1). The larger family (GP2-FAM-1) included four affected individuals showing the segregation of this variant with PD. The second family (AMP-FAM-1) consisted of three carriers, one clinically affected with PD and two asymptomatic carriers (at ages 55 and 76 years, respectively). The remaining two carriers were PD cases with a positive family history of PD, but no additional family members were available for genetic testing. Notably, rs111910483 is multiallelic, and we identified 7 additional carriers of the synonymous p.L1795L (ENST00000298910.12:c.5385 G > A; chr12:40322386:G:A) variant. However, this synonymous variant is very unlikely to be disease-causing and was therefore excluded from any further analyses. Additionally, we did not identify other recurrent variants in known PD genes with supporting segregation evidence.

**Fig. 1:**
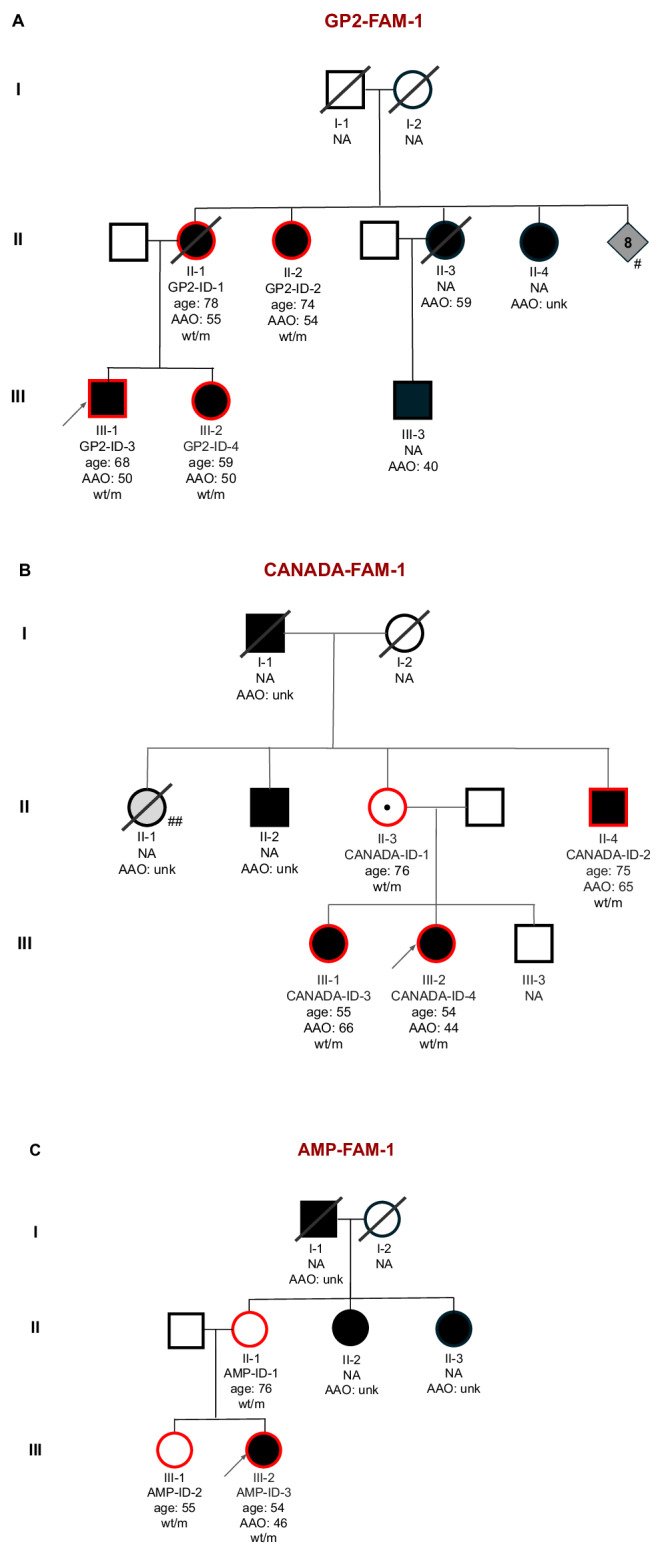
Pedigrees of identified families in this study. Pedigree of Family GP2-FAM-1 (A), CANADA-FAM-1 (B), and AMP-FAM-1 (C) with the *LRRK2* p.L1795F variant. The pedigrees were drawn based on reported family history and may be incomplete. The index cases are indicated with arrows. Affected individuals are indicated by black symbols: circles (female) and squares (male). Diamond is where sex is undefined. Unaffected individuals are indicated by open symbols. Unaffected variant carriers are indicated by open symbols with a dot in the middle. A diagonal line indicates deceased individuals. Red circle indicates individuals with genetic data available (WGS data for GP2-FAM-1 and AMP-FAM-1, single gene testing for CANADA-FAM-1). Heterozygous mutant (m) and wild-type (wt) genotypes are indicated with corresponding age at the sample collection (age) and age at motor symptom onset (if known; AAO). **A** The mother of GP2-FAM-1 index was reported to have eight additional siblings (#), several of whom are clinically affected with PD; however, no detailed family history is available for these relatives. **B** One maternal aunt (II-1) of the CANADA-FAM-1 index was reported to have had Alzheimer’s disease (##).

Next, we screened the genotyping data of 54,153 affected and unaffected individuals generated within GP2 (DOI: 10.5281/zenodo.10962119), where the *LRRK2* p.L1795F variant was directly genotyped using the Neurobooster array. We identified three additional clinically affected variant carriers (Supplementary Fig. 7). We further screened the clinical exome data from 10,454 individuals from PDGENE which resulted in one additional variant carrier (Supplementary Fig. 8). Finally, querying the CENTOGENE proprietary Databank CentoMD®^12^, we identified another family with four individuals carrying the *LRRK2* p.L1795F variant, three of whom were PD cases and one being an asymptomatic carrier. In total, we identified 17 individuals carrying this variant across all the datasets, including nine index cases with PD as well as five affected and three unaffected family members.

The demographic and clinical details of all identified variant carriers are displayed in Table 1. More than two-thirds were females (70.6%; *n* = 12/17). All affected and unaffected carriers had a positive family history of PD. Notably, among the six singleton cases, two reported only second-degree relatives with PD, while three reported a multi-incident family history of the disease. Ages of motor symptom onset (AAO) in affected individuals ranged from 36 to 66 years. The median AAO was 54.5 years (interquartile range 47-60 years). The asymptomatic carriers were 55, 76 and 76 years old, respectively, at the time of sample collection and clinical evaluation. Based on the available clinical data, the majority of affected individuals had classical PD with an asymmetric onset of symptoms and a good response to dopaminergic medication, and without obvious atypical signs suggestive of other diagnoses (missing data for up to 30%). Detailed data on non-motor symptoms and neuropsychiatric comorbidities were scarce. Cognition was reported to be unaffected in the majority of affected carriers with good scores in cognition tests (including Montreal Cognitive Assessment [MoCA] and Mini Mental State Examination [MMSE]); however, one clinically affected individual had significant cognitive impairment (MoCA score of 17 points) and one unaffected carrier also showed some cognitive deficits (MoCA score of 23 points). More detailed characteristics of the individuals from the three identified families are available in the Supplementary Material.

**Table 1:** Demographic and clinical characteristics of identified *LRRK2* p.L1795F variant carriers

Cohort	GP2								AMP-PD				PDGENE	CANADA			
Family ID	GP2-FAM-1				NA	NA	NA	NA	AMP-FAM-1					CANADA-FAM-1			
Sample ID	**GP2-ID-1**	**GP2-ID-2**	**GP2-ID-3**	**GP2-ID-4**	**GP2-ID-5**	**GP2-ID-6**	**GP2-ID-7**	**GP2-ID-8**	**AMP-ID-1**	**AMP-ID-2**	**AMP-ID-3**	**AMP-ID-4**	**PDGENE-ID-1**	**CANADA-ID-1**	**CANADA-ID-2**	**CANADA-ID-3**	**CANADA-ID-4**
Genetic method	NBA, WGS	NBA, WGS	NBA, WGS	NBA, WGS	NBA, WGS	NBA	NBA	NBA	WGS	WGS	WGS	WGS	CES	Single gene testing (*LRRK2*)			
**Demographics**																	
Gender	Female	Female	Male	Female	Male	Male	Female	Male	Female	Female	Female	Female	Female	Female	Male	Female	Female
Genetic ancestry	EUR	EUR	EUR	EUR	EUR	EUR	EUR	EUR	EUR	EUR	EUR	EUR	EUR	White	White	White	White
Age at sample collection	78	74	66	68	42	72	62	76	76	55	54	69	57	76	75	55	54
Family history of PD	yes	yes	yes	yes	yes	yes	yes	yes	yes	yes	yes	yes	yes	yes	yes	yes	yes
Family history details	two children, three sisters, one nephew, several aunts and uncles	three sisters, one niece and two nephews, several aunts and uncles	sister, mother, three maternal aunts	brother, mother, three maternal aunts	aunt, two great uncles	mother, brother	mother, sister	mother	father, two siblings, child	sibling, maternal grand- parent, maternal aunt	maternal grand- prarent, two maternal aunts	mother	maternal grand- mother	father, two siblings, two children	father, two siblings, two nieces	sibling, two maternal uncles, maternal grandfather	sibling, two maternal uncles, maternal grandfather
**Clinical data**																	
Diagnosis	PD	PD	PD	PD	PD	PD	PD	PD	Control*	Control*	PD*	PD	PD	Control**	PD	PD	PD
AAO	55	54	58	50	36	60	57	55	NA	NA	46	65	47	NA	65	66	44
AAE	78	74	67	68	42	72	62	76	76	55	54	69	57	76	75	67	66
Bradykinesia	+	+	+	+	+	+	+	+	NA	NA	+	+	+	-	+	+	+
Rigidity	+	+	+	+	+	-	+	+	NA	NA	+	+	+	-	-	+	+
Resting Tremor	+	+	+	+	+	+	-	+	NA	NA	+	+	-	-	+	-	-
Action/Kinetic Tremor	+	+	+	+	-	+	+	NA	NA	NA	-	+	-	-	-	+	+
Postural Instability	+	+	-	+	+	-	+	+	NA	NA	-	-	-	-	-	-	+
Gait Disturbance	+	+	-	+	+	-	-	NA	NA	NA	-	+	-	-	-	-	+
Asymmetric onset of symptoms	+	+	+	+	+	+	+	NA	NA	NA	+	NA	+	-	+	-	+
Responsive to dopaminergic medication	+	+	+	+	+	+	+	NA	NA	NA	+	NA	+	NA	NA	NA	+
Fluctuations	NA	NA	+	+	-	NA	NA	NA	NA	NA	+	NA	+	-	-	-	+
UPDRS Part III (motor score)	70	NA	10	22	24	6	11	NA	NA	NA	3	32	6	0	6	7	43
Hoehn & Yahr	5	2	2	2	2	1	1.5	NA	NA	NA	2	2	2	0	1	0	3
Cognition	MMSE 29	MMSE 29	MMSE 30	MMSE 30	MMSE 30	MMSE 30	MMSE 30	NA	NA	NA	MoCA 28	NA	-	MoCA 23	MoCA 17	MoCA 29	MoCA 28
Neuro-psychiatric features	NA	NA	-	-	NA	NA	NA	NA	NA	NA	NA	NA	-	NA	NA	-	+
Dysautonomia	-	-	-	constipation	-	-	-	NA	NA	NA	NA	NA	-	-	-	-	-
Atypical Features or signs suggestive of other diagnosis (#)	history of head trauma with loss of concious-ness	-	-	-	history of head trauma with loss of concious-ness	-	-	NA	NA	NA	NA	NA	-	-	-	-	-

+present; -absent.

*AAE* age at clinical examination, *AAO* age at motor symptom onset, *EUR* European, *MMSE* Mini Mental State Examination, *MOCA* Montreal Cognitive Assessment, *NA* Not available or applicable, *NBA* NeuroBooster Array, *PD* Parkinson’s disease, *CES* clinical-exome sequencing, *WGS* Whole-genome sequencing.

*Individuals were recruited through the LCC as “Genetically enriched” study arm.

**Recruited as unaffected family member, not population control.

(#) These include: history of strokes or stepwise deterioration, history of head injury with loss of consciousness, history of encephalitis, Oculogyric crisis, neuroleptic treatment at time of symptom onset, sustained remission, gaze palsy, Cerebellar signs (other than activation tremor), Fluctuations, hallucinations, dysautonomia, Memory loss, or prominent axial rigidity.

The p.L1795F (ENST00000298910.12:c.5385 G > T) variant is currently categorized as a variant of uncertain significance in ClinVar and shows conflicting evidence from various *in-silico* prediction tools and databases (Supplementary Table 2 and Supplementary Fig. 9). It is rare and confined to European populations in several investigated databases (including gnomAD v4.1, the Regeneron Genetics Center Million Exome Variant Browser^13^, and the UK Biobank^14^ 500 K genomes). Similarly, all identified *LRRK2* p.L1795F carriers in this study were of European ancestry, whereas the variant was absent in other ancestral populations (*n* = 15,316) within the GP2 genotyping cohort. In Europeans, it had an allele frequency of 0.00012 among PD cases (5 heterozygous carriers and 20,812 noncarriers) while being absent in controls (*n* = 9,032; Table 2). The logistic regression analysis using the European population of the GP2 genotyping cohort did not reveal a significant association between this variant and PD, likely due to insufficient controls available in the dataset given its rarity (*P* > 0.8, Supplementary Table 3). When comparing the distribution of carriers between PD cases from the combined genotyping and WGS dataset (6 heterozygous carriers and 23,270 noncarriers) and two non-Finnish European control populations: gnomAD v3.1.2 non-neuro (0 heterozygous carriers and 31,960 noncarriers) and gnomAD v4.1 (2 heterozygous carriers and 589,826 noncarriers), this variant was significantly associated with PD (*P* < 0.0056 using gnomAD v3.1.2 non-neuro, and *P* < 7.84e-08, OR = 76.04, 95% CI: 15.35–376.77 using gnomAD v4.1, two-tailed Fisher’s exact test). Given this variant was observed only in the European population, we searched for the overlapping IBD segments among the variant carriers using the genotyping data. The median length of an IBD segment over *LRRK2* in these individuals was 7.05 cM (range: 2.1–96.3 cM, Fig. 2). All genotyped carriers shared a core haplotype of 2.825 Mbp at this locus (Supplementary Table 4), suggesting that the p.L1795F variant descended from a common founder.

**Table 2:** Frequency of the *LRRK2* p.L1795F and p.G2019S variants across ancestries in the GP2 genotyping cohort

Variant	Ancestry	AF in cases (allele count)	AF in controls (allele count)	Number of alleles in cases	Number of alleles in controls
**chr12:40322386:G:T (*LRRK2*** **p.L1795F)**	EUR	0.0001201 (5)	0 (0)	41634	18064
**chr12:40340400:G:A (*LRRK2*** **p.G2019S)**	AAC	0 (0)	0.0006281 (1)	568	1592
	AFR	0 (0)	0 (0)	1876	3252
	AJ	0.07081 (181)	0.01098 (9)	2556	820
	AMR	0.01339 (12)	0.003247 (1)	896	308
	CAH	0.006783 (7)	0.003436 (2)	1032	582
	CAS	0 (0)	0 (0)	1104	688
	EAS	0 (0)	0 (0)	5122	4752
	EUR	0.003266 (136)	0.000166 (3)	41636	18074
	FIN	0 (0)	0 (0)	192	14
	MDE	0.02805 (17)	0 (0)	606	446
	SAS	0 (0)	0 (0)	732	412

*AF* Allele frequency, *AAC* African admixed, *AFR* African, *AJ* Ashkenazi Jewish, *AMR* Latino and Indigenous people of the Americas, *CAH* Complex Admixture History, *CAS* Central Asian, *EAS* East Asian, *EUR* European, *FIN* Finnish, *MDE* Middle Eastern, *SAS* South Asian.

*LRRK2*: ENST00000298910.12; ENSP00000298910.7.

**Fig. 2:**
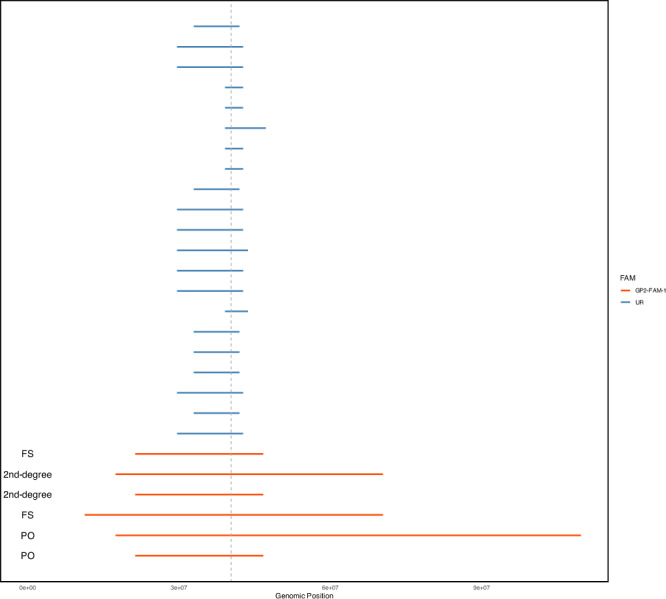
Overlapping identity-by-descent segments spanning *LRRK2* p.L1795F variant among the variant carriers with genotyping data. Each line represents an IBD segment inferred between a unique pair of individuals. IBD segments are colored based on whether both individuals in a pair belong to the same family (GP2-FAM-1) or are considered unrelated (UR). FS indicates an IBD segment between full siblings, 2nd degree refers to a segment between a pair of second-degree relatives, and PO represents a segment between a parent and offspring. The vertical grey line marks the genomic position of the *LRRK2* p.L1795F variant.

To our knowledge, we provide the largest number of *LRRK2* p.L1795F variant carriers thus far, including 14 carriers clinically affected with PD and three asymptomatic carriers. The available data from the previously reported carriers^5–8^ do not align with our data, making an overlap of individuals between the studies unlikely. Including those reported in the literature, this brings the total to 22 clinically affected carriers of European ancestry. Still, the overall number of p.L1795F carriers is limited, and higher frequencies might be observed in specific European subpopulations. Our haplotype analysis indicating a common founder further supports this hypothesis, although we were only able to determine the geographical origin of one family of carriers in this study, which was of Ukrainian and Polish descent. Taken together with four recently published carriers of either Hungarian or Slovak origin, this likely indicates a Central-Eastern European origin^8^. Notably, we identified three asymptomatic p.L1795F carriers, who might still develop PD symptoms later in life. However, given the pedigree structure of these individuals, this may also reflect reduced penetrance - a common phenomenon in monogenic forms of PD, including other pathogenic *LRRK2* variants.

Comparing the clinical phenotypes of p.L1795F carriers with those of other pathogenic *LRRK2* variants, particularly p.G2019S^15^, revealed similarities among them and with idiopathic PD (iPD). While group differences in clinical phenotypes among *LRRK2* variants may exist^16^, they do not enable meaningful genotype-phenotype correlations at an individual level. LRRK2-PD is clinically indistinguishable from iPD on an individual level. Most individuals with LRRK2-PD, including p.L1795F carriers, exhibit a classic PD phenotype with a good response to dopaminergic treatment. Atypical presentations have been described in single cases but are overall rare^16^. Notably, the p.L1795F variant is located in the COR-B domain, in close proximity to other pathogenic *LRRK2* variants, namely p.Y1699C^17^ and p.F1700L^18^. Interestingly, for p.Y1699C carriers, a more heterogeneous phenotype has been reported, including atypical signs like amyotrophy, dementia and symptoms of behavioral disorders.^17,19–21^ However, this observation might be coincidental and biased by the small number of variant carriers. Atypical features, prominent non-motor features, or neuropsychiatric comorbidities haven’t been specifically reported for the majority of p.L1795F carriers, but the overall data is limited, making it difficult to draw meaningful conclusions. Overall, the p.L1795F phenotype aligns well with the general characteristics of LRRK2-PD and appears comparable to other *LRRK2* variants with cautious interpretation given the limited number of identified carriers. The most significant differences between the genetic subtypes are their ancestral and geographical variability.

In conclusion, this is the first study providing evidence of the *LRRK2* p.L1795F variant segregating with disease in multiplex families, missing from the previous reports^5–8^. Taken together with published functional data^4^, showing strongly enhanced LRRK2 kinase activity, our findings support the *LRRK2* p.L1795F variant to be considered pathogenic. Large-scale studies can be helpful to identify novel rare causes of PD but also to re-evaluate previously identified variants by providing additional evidence of pathogenicity through an increased number of variant carriers and segregation. We therefore propose *LRRK2* p.L1795F as a cause of PD, especially in the European population. Including this variant in the genetic screening of PD patients, particularly those of Central-Eastern European origin, may be beneficial for the variant carriers to be included in ongoing gene-specific clinical trials.

## Methods

### Ethics declaration

This study was conducted in accordance with the ethical standards of the institutional and national research committees. This study was approved by all ethics committees or institutional review boards of all sites participating in this study and providing samples and data, including the University of Cincinnati in Cincinnati (IRB#2017-5985), Ohio, USA, the Emory University School of Medicine in Atlanta, GA, USA, and the Michigan State University, MI, USA, and the University Health Network Research Ethics Board in Toronto, Canada. Informed consent for study participation was obtained from all participants.

### Study design and participants

**Fig. 3:**
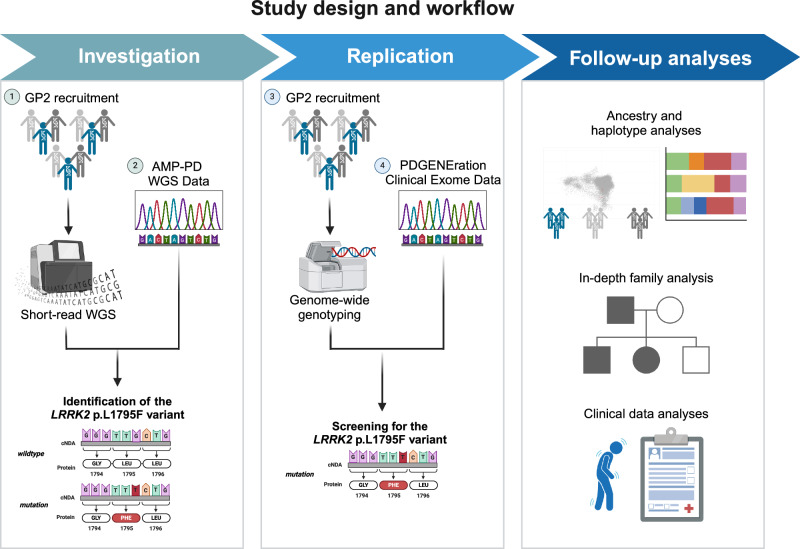
Study design. Figure created with BioRender.com.

Our study workflow is highlighted in Fig. 3. Three sources of data were included in this study (Supplementary Table 1). First, we used the multi-ancestry whole-genome sequencing and genotyping data from the study participants recruited as part of GP2^22^ (DOI 10.5281/zenodo.13755496) as previously described^23,24^. Individual-level demographic and clinical data were obtained from participating principal investigators and publicly available databases (e.g., for Coriell samples included in GP2). Second, we incorporated whole-genome sequencing data from AMP-PD. Participants in this initiative were recruited through multiple studies, including BioFIND, the Harvard Biomarkers Study (HBS), the Lewy Body Dementia Case-Control Cohort (LBD), the Parkinson’s Disease Biomarkers Program (PDBP), the Parkinson’s Progression Markers Initiative (PPMI), the LRRK2 Cohort Consortium (LCC), the Study of Isradipine as a Disease-Modifying Agent in Subjects with Early Parkinson Disease, Phase 3 (STEADY-PD3), and the Study of Urate Elevation in Parkinson’s Disease, Phase 3 (SURE-PD3). Clinical information and genetic samples from participants were obtained with appropriate written consent and local institutional and ethical approvals. Detailed information about these studies is available on the AMP-PD website (https://amp-pd.org) and the respective study websites. Third, we obtained the clinical exome sequencing data from PDGENE^2^, a large multi-center study in North America providing genetic testing and counseling to more than 15,000 participants.

### Whole-genome sequencing (WGS) data

We included 9974 samples with the sequence alignment data available from BioFIND, HBS, LBD, PDBP, PPMI, STEADY-PD3, and SURE-PD3 cohorts through the AMP-PD release for joint genotyping with the GP2 cohort (Supplementary Table 5). Due to the unavailability of sequence alignment data from the LCC cohort, we used AMP-PD release 4 data to screen for potential pathogenic variants in this cohort.

Additionally, the DNA samples from 5,926 participants from the GP2 cohort (GP2 Data Release 8, DOI 10.5281/zenodo.13755496, Supplementary Table 5) were genome sequenced to an average of 30x coverage with 150 bp paired-end reads following Illumina’s TruSeq PCR-free library preparation protocol. We followed the same functional equivalence pipeline^25^ as AMP-PD to produce the sequence alignment against the GRCh38DH reference genome.

We used DeepVariant v.1.6.1^26^ (https://github.com/google/deepvariant) to generate the single-sample variant calls for a total of 15,900 samples in GP2 and AMP-PD and performed joint-genotyping using GLnexus v1.4.3 (https://github.com/dnanexus-rnd/GLnexus) with the preset DeepVariant WGS configuration^27^. We set genotypes to be missing after variant quality control defined as genotype quality >=10, read depth >=10, and heterozygous allele balance between 0.2 and 0.8, and retained high-quality variants with a call rate > 0.95 after quality control. After the sample quality control following the quality metrics defined by AMP-PD^28^, we retained 15,752 samples (AMP-PD and GP2 combined) for the downstream analyses (Supplementary Table 5). Variant annotation was performed with Ensembl Variant Effect Predictor v111 (http://www.ensembl.org/info/docs/tools/vep/index.html, RRID:SCR_007931)^29^. We used KING v.2.3.0 (https://www.kingrelatedness.com, RRID:SCR_009251)^30^ to infer relatedness up to the second-degree relatives to confirm the known relationships and identify cryptic familial relationships. Genetic ancestry was determined using GenoTools v1.2.3 (https://github.com/GP2code/GenoTools) with the default settings^31^.

### Genome-wide genotyping with the Neurobooster Array (GP2)

We screened the genotyping data published as part of GP2’s Data Release 7^32^ (DOI: 10.5281/zenodo.10962119, Supplementary Table 6). Genotyping was performed by GP2 using the NeuroBooster Array (NBA; v.1.0, Illumina, San Diego, CA)^33^. Raw genotyping data underwent quality control and genetic ancestry prediction using GenoTools v1.2.3 with the default settings^31^. The *LRRK2* p.L1795F variant was directly genotyped using NBA, and the quality of genotype calls was assessed by examining the signal intensity plots.

### Clinical exome sequencing (PDGENEration)

We included 10,454 samples with clinical exome data available from PDGENE^2^ as part of GP2’s Data Release 8 (DOI 10.5281/zenodo.13755496)^32^. The sequence data processing followed the same pipeline of WGS data as mentioned above. We performed joint-genotyping using GLnexus v1.4.3 with the preset DeepVariant WES configuration and followed the same criteria for sample and variant quality control as for the WGS data.

### Querying additional databases (CENTOGENE)

We queried the CENTOGENE proprietary Databank CentoMD®^12^ to identify potential additional variant carriers. CENTOGENE is a globally operating genetic diagnostic lab. Genetic data included in this manuscript was generated by exon-wise PCR amplification followed by Sanger sequencing.

### Statistical analyses

To estimate the allele frequency of *LRRK2* p.L1795F variant in multi-ancestral populations, we analyzed the GP2 genotyping data, the largest available dataset in this study. We excluded related individuals and samples from targeted recruitment, such as *LRRK2* and *GBA1* variant carriers within specific efforts of PPMI and LCC. Subsequently, we performed an association analysis of this variant with PD using the European population. We fitted the logistic regression model with PD status as binary outcome variable and the covariates as the genotype of *LRRK2* p.L1795F variant, sex, age, family history, and the first six principal components to account for the population stratification. For cases, age at onset (AAO) or age at diagnosis was used, while for controls, age at sampling was used. Additionally, we merged GP2 genotyping data with the combined AMP-PD and GP2 WGS data, resulting in a cohort of 23,276 PD cases of European ancestry after excluding duplicated, related, and targeted recruitment samples as mentioned above. This allowed us to compare the carrier distribution between PD cases and non-Finnish European population from the Genome Aggregation Database (gnomAD v.3.1.2 non-neuro and v4.1, http://gnomad.broadinstitute.org/, RRID:SCR_014964) as external population controls using Fisher’s exact test. We excluded the PDGENE clinical exome data from this analysis as we could not estimate the genetic ancestry in the same manner as with the other datasets. The *P* value ≤ 0.05 was considered statistically significant for all the analyses.

To determine if carriers of the *LRRK2* p.L1795F variant shared recent common ancestry, we phased the genotyping data from chromosome 12 in the European population using Beagle 5.4 (https://faculty.washington.edu/browning/beagle/beagle.html) with default settings^34^ and searched for identical-by-descent (IBD) segments with the length ≥2 cM shared across the carriers using hap-ibd v1.0.0 (https://github.com/browning-lab/hap-ibd) with default setting^35^.

## Supplementary information

Supplementary Material

## Data Availability

GP2 partnered with the online cloud computing platform Accelerating Medicines Partnership - Parkinson’s Disease (AMP PD; https://amp-pd.org) to share data generated by GP2. Qualified researchers are encouraged to apply for direct access to the data through AMP PD. The GP2 and AMP-PD datasets analysed during the current study are available through AMP-PD (https://amp-pd.org). Additional data analysed during this study (Centogene) are included in this published article.

## Code availability

All scripts used for this study can be found in the public domain on GitHub (https://github.com/GP2code/EUR_LRRK2_pL1795F).
