# Supplementary material for: The LRRK2 p.L1795F variant causes Parkinson’s disease in the European population
Source: NPJ Parkinsons Dis. 2025 Mar 25;11:58. doi: 10.1038/s41531-025-00896-2 (PMC11937388; doi:10.1038/s41531-025-00896-2)
Supplement: Supplementary file 1 — Supplementary Material [file 41531_2025_896_MOESM1_ESM.pdf]

Supplementary Material

The *LRRK2* p.L1795F variant causes Parkinson's disease in the European population

Lara M. Lange,[1,2,3] Kristin Levine,[4,5] Susan H. Fox,[6] Connie Marras,[6] Nazish Ahmed,[6] Nicole Kuznetsov,[4,5] Dan Vitale,[4,5] Hirotaka Iwaki,[3,4,5] Katja Lohmann,[1] Luca Marsili,[7] Alberto J. Espay,[7] Peter Bauer,[8] Christian Beetz,[8] Jessica Martin,[5] Stewart A. Factor,[9] Lenora A. Higginbotham,[9] Honglei Chen,[10] Hampton Leonard,[4,5] Mike Nalls,[4,5] Niccolo E. Mencacci, PhD,[11] Huw R. Morris,[12,13] Andrew Singleton,[3,5] Christine Klein,[1,2] Cornelis Blauwendraat,[3,5] Zih-Hua Fang,[14] and *the Global Parkinson's Genetics Program (GP2)*

Affiliations:

[1] Institute of Neurogenetics, University of Luebeck, Luebeck, Germany
[2] Department of Neurology, University Hospital Schleswig-Holstein, Luebeck, Germany
[3] Laboratory of Neurogenetics, National Institute on Aging, National Institutes of Health, Bethesda, MD, USA
[4] DataTecnica, Washington, DC, USA
[5] Center for Alzheimer's and Related Dementias (CARD), National Institute on Aging and National Institute of Neurological Disorders and Stroke, National Institutes of Health, Bethesda, MD, USA
[6] Edmond J. Safra Program in Parkinson's Disease and the Morton and Gloria Shulman Movement Disorders Clinic, Toronto Western Hospital, University Health Network, University of Toronto, Toronto, Ontario, Canada.
[7] University of Cincinnati, Cincinnati, Ohio, USA
[8] CENTOGENE GmbH, Am Strande 7, 18055 Rostock, Germany
[9] Department of Neurology, Emory University School of Medicine, Atlanta, GA, USA
[10] Department of Epidemiology and Biostatistics, Michigan State University, MI, USA
[11] Department of Neurology, Northwestern University Feinberg School of Medicine, Chicago, IL, USA
[12] Department of Clinical and Movement Neurosciences, UCL Queen Square Institute of Neurology, London, UK
[13] UCL Movement Disorders Centre, University College London, London, UK
[14] German Center for Neurodegenerative Diseases (DZNE), Tübingen, Germany

Detailed clinical synopsis for GP2-FAM1, CANADA-FAM1, and AMP-FAM-1

Family GP2-FAM-1

Family GP2-FAM-1 is of European ancestry with Ukrainian and Polish origin. Seven individuals are known to be clinically affected by PD, including the index patient (GP2-ID-3), his sister, mother, three maternal aunts, and a maternal cousin, consistent with autosomal dominant inheritance (Figure 2). Further, additional maternal aunts and uncles were reported to have PD but a detailed history was not available. We identified the *LRRK2* p.L1795F variant segregating within all four tested family members from both NBA and WGS data. No unaffected family samples were available. Screening variants segregating within this family from the WGS data did not reveal any other potential causal variants, including known pathogenic variants in the established dominant PD genes *SNCA* and *VPS35* as well as other variants in *LRRK2* and pathogenic *GBA1* variants.

All family members with available data were reported to have bradykinesia, rigidity, resting and action tremor, and motor symptoms that were responsive to dopaminergic treatment. Disease progression was mild to moderate in three of four individuals with low to moderate UPDRS (part III) motor scores and a Hoehn & Yahr stage 2 after 9+ years of disease duration. Only one individual, GP2-ID-1 (deceased), seemed to have had a more progressive disease course with a high UPDRS (part III) motor score and Hoehn & Yahr stage 5, though over a disease duration of more than 20 years. Neuropsychiatric comorbidities or severe autonomic features were not reported in those with available data. Cognition was unaffected in all family members. All but one affected family member, including those without genetic testing, had an AAO in their fifties (ranging from 50 to 59 years), and only one individual had a lower AAO of 40 years.

Family CANADA-FAM-1

Family CANADA-FAM-1 included four individuals available for genetic testing, and all four carried the *LRRK2* p.L1795F variant. The index case as well as her sister and a maternal uncle were clinically affected with PD whereas the mother of both siblings was an unaffected carrier. There were additional family members clinically affected with PD, including another maternal uncle and the maternal grandfather, both of which were unavailable for genetic testing within this study (Figure 2).

The reported AAO of the index case was 44 years and thereby younger than for the other two tested family members, which were 65 and 66, respectively. The index patient and her sister have been followed up for almost 12 years, whereas the other two individuals (CANADA-ID-1 and CANADA-ID-2) were only clinically assessed once in 2012. Notably, the index patient had a more progressive PD disease course than her sister, as indicated by a higher UPDRS motor score of 43 points and Hoehn & Yahr stage 3, compared to only 7 points in the UPDRS (part III) in her sister. All three affected individuals had a diagnosis of classical PD without any atypical features; however, one individual (CANADA-ID-1) had significant cognitive impairment with a low MoCA score of only 17 points. Interestingly, also the unaffected carrier showed some cognitive impairment (MoCA score of 23 points) but no motor symptoms of PD nor any other prodromal signs of disease.

Family AMP-FAM-1

Family AMP-FAM-1 included three individuals available for genetic testing, all of whom carried the p.L1795F variant. The index case was clinically affected by PD, while her sister and mother were both asymptomatic. The family history of PD was strongly positive, with multiple additional affected family members, including two maternal aunts and the maternal grandfather of the index, suggesting autosomal dominant inheritance with reduced penetrance (Figure 3). The index patient had a reported AAO of 46 years and a very low UPDRS (part III) motor score, indicating a rather mild disease course. Clinical details for the additional affected family members were unavailable. The two asymptomatic carriers were 55 and 76 years old at sample collection and showed no motor signs of PD. Details on the presence or absence of prodromal signs were not available. We did not identify other potential disease-causing variants in this family by WGS.

Supplementary Tables

Supplementary Table 1: Overview of the investigated cohorts

Cohort	Investigation		Replication	
	**GP2**	**AMP-PD [3]**	**GP2**	**PDGENE**
**Data type**	WGS	WGS	NBA	CES
**Total number of samples**	5,796	9,956 (599)	54,180	10,454
**PD cases**	5,283	3,442	28,729	10,454
**Other phenotypes [1]**	161	2,903	15,834	NA
**Controls [2]**	342	4,210	9,617	NA

AMP-PD = Accelerating Medicines Partnership Parkinson's disease, GP2 = Global Parkinson's Genetics Program, NA = not available, NBA = NeuroBooster Array, PD = Parkinson's disease, PD GENE = PD GENEration study, CES = clinical-exome sequencing, WGS = whole-genome sequencing

[1] Other phenotypes include atypical parkinsonism, e.g., progressive supranuclear palsy (PSP), multi system atrophy (MSA), corticobasal degeneration/syndrome (CBD/CBS), and dementia with Lewy bodies (DLB), as well as prodromal PD.
[2] Controls include asymptomatic carriers of known pathogenic variants.
[3] Joint-genotyping using the 9,956 samples from BioFIND, Harvard Biomarkers Study (HBS), Lewy body dementia case-control cohort (LBD), Parkinson's disease Biomarkers Program (PDBP), Parkinson's Progression Markers Initiative (PPMI), Study of Isradipine as a Disease-modifying Agent in Subjects With Early Parkinson Disease, Phase 3 (STEADY-PD3), Study of Urate Elevation in Parkinson's Disease, Phase 3 (SURE-PD3), and Postmortem Cohort. AMP-PD release 4 was used to screen for potential pathogenic variants for the 599 samples from the LRRK2 Cohort Consortium (LCC).

Supplementary Table 2. Evaluation of the pathogenic role of the *LRRK2* p.L1795F variant using ACMG criteria

Domain	Supporting	Not supporting	Source	ACMG criteria
**Frequency**	Rare and confined to the European population<br>gnomAD v4 MAF overall: 0.000001239<br>gnomAD v4 MAF European (non-Finnish): 0.000001695		gnomAD	PM2
**In silico prediction tools [1]**	VEST4 (0.928)<br>MutationTaster (Disease-causing)	CADD (19.94)<br>REVEL (0.638)<br>ClinVar (Uncertain significance)<br>Franklin (VUS)<br>Varsome (Uncertain significance)	Pejaver et al., 2022	
**Conservation**	Highly conserved across species		MutationTaster	
**Functional evidence**	Strongly enhancing LRRK2 kinase activity [2]		Kalogeropulou et al., 2022	PS3
**Literature**	Proposed as a strong risk factor (OR 2.5) [3]	Only single case reports, no evidence of segregation [4-7]	Pitz et al., 2024<br>Nichols et al., 2007<br>Benitez et al., 2016<br>Illés et al., 2019<br>Ostrozovicova et al., 2024	PM*
**Segregation**	Variant segregating with the disease in two multiplex families		This study	PP1**

OR = Odds ratio, VUS = variant or uncertain significance
* Observation of the variant in multiple unrelated individuals with the same phenotype (no specific criterion, may be considered as moderate evidence)
** Strong evidence of segregation (based on our findings upgraded to strong evident by segregation in three families with two generations of family members each)

References

1. Pejaver, V. *et al.* Calibration of computational tools for missense variant pathogenicity classification and ClinGen recommendations for PP3/BP4 criteria. *Am. J. Hum. Genet.* **109**, 2163–2177 (2022).
2. Kalogeropulou, A. F. et al. Impact of 100 LRRK2 variants linked to Parkinson's disease on kinase activity and microtubule binding. Biochem. J 479, 1759–1783 (2022).
3. Pitz, V. et al. Analysis of rare Parkinson's disease variants in millions of people. NPJ Parkinsons Dis 10, 11 (2024).
4. Nichols, W. C. *et al.* LRRK2 mutation analysis in Parkinson disease families with evidence of linkage to PARK8. *Neurology* **69**, 1737–1744 (2007).
5. Benitez, B. A. *et al.* Resequencing analysis of five Mendelian genes and the top genes from genome-wide association studies in Parkinson's Disease. *Mol. Neurodegener.* **11**, 29 (2016).
6. Illés, A. *et al.* The Role of Genetic Testing in the Clinical Practice and Research of Early-Onset Parkinsonian Disorders in a Hungarian Cohort: Increasing Challenge in Genetic Counselling, Improving Chances in Stratification for Clinical Trials. *Front. Genet.* **10**, 1061 (2019).
7. Ostrozovicova, M. *et al.* p.L1795F LRRK2 variant is a common cause of Parkinson's disease in Central Europe. *Res. Sq.* (2024) doi:10.21203/rs.3.rs-4378197/v1.

Supplementary Table 3. Results of logistic regression of *LRRK2* p.L1795F variant and PD status

Variant	REF	ALT	A1_FREQ	FIRTH?	TEST	OBS_CT	OR	LOG(OR)_SE	L95	U95	Z_STAT	P
***LRRK2* p.L1795F**	G	T	0.000154454	Y	ADD	16186	0.711377	1.48804	0.0385012	13.1439	-0.22886	0.818978
***LRRK2* p.L1795F**	G	T	0.000154454	Y	PC1	16186	0.969697	0.0237548	0.925584	1.01591	-1.29539	0.195185
***LRRK2* p.L1795F**	G	T	0.000154454	Y	PC2	1.62E+04	0.791958	0.0269728	0.751178	0.834952	-8.64748	5.27E-18
***LRRK2* p.L1795F**	G	T	0.000154454	Y	PC3	1.62E+04	0.842527	0.0304225	0.793758	0.894292	-5.63234	1.78E-08
***LRRK2* p.L1795F**	G	T	0.000154454	Y	PC4	1.62E+04	1.1674	0.0251755	1.11119	1.22645	6.14798	7.85E-10
***LRRK2* p.L1795F**	G	T	0.000154454	Y	PC5	1.62E+04	1.13268	0.0246203	1.07932	1.18868	5.06035	4.18E-07
***LRRK2* p.L1795F**	G	T	0.000154454	Y	PC6	16186	1.0133	0.0262801	0.962425	1.06686	0.502614	0.615235
***LRRK2* p.L1795F**	G	T	0.000154454	Y	SEX	1.62E+04	0.73539	0.0196464	0.707611	0.764259	-15.6443	3.63E-55
***LRRK2* p.L1795F**	G	T	0.000154454	Y	AGE	16186	5.02E-01	0.0239323	0.478618	0.525693	-28.829	9.29E-183

***LRRK2* p.L1795F**	G	T	0.000154454	Y	Family_History	16186	1.53E+00	0.0236682	1.46112	1.60317	17.9817	2.71E-72

Supplementary Table 4: The common haplotype shared by *LRRK2* p.L1795F (12-40322386-G-T) carriers inferred from the Neurobooster array

rsID	Chr-Pos-Ref-Alt	GP2 MAF*	gnomAD# EUR alternative AF	GP2-ID-9 hap1	GP2-ID-2 hap1	GP2-ID-1 hap1	GP2-ID-4 hap1	GP2-ID-5 hap1	GP2-ID-3 hap2	GP2-ID-8 hap2	GP2-ID-7 hap2
rs4768592	12-39150187-T-C	0.376323	0.3733	C	C	C	C	C	C	C	C
rs7980466	12-39246948-G-A	0.213643	0.2118	A	A	A	A	A	A	A	A
rs11171902	12-39376846-C-T	0.0564491	0.05641	C	C	C	C	C	C	C	C
rs55988438	12-39603259-G-A	0.0355401	0.03401	A	A	A	A	A	A	A	A
rs11174343	12-39930504-T-C	0.151409	0.1485	C	C	C	C	C	C	C	C
rs7971218	12-40066725-A-G	0.165508	0.1596	G	G	G	G	G	G	G	G
rs111910483	12-40322386-G-T	0.00010377	1.70E-06	T	T	T	T	T	T	T	T
rs11564252	12-40419931-T-C	0.186469	0.1784	C	C	C	C	C	C	C	C
rs11179176	12-40953962-A-G	0.33051	0.3276	G	G	G	G	G	G	G	G

rs3906837	12-41723638-T-C	0.344557	0.349	C	C	C	C	C	C	C	C

AF = allele frequency, EUR = European, MAF = minor allele frequency

* GP2 MAF was calculated based on the GP2 European genotyping cohort.
# The gnomAD frequency is based on gnomAD version v4.1.

Supplementary Table 5: Study participant demographic information for whole-genome sequencing data from the combined GP2 and AMP-PD datasets

**Ancestry**	**Total**	**PD**	**Controls**	**Other phenotypes [1]**
African	258	158	95	5
African Admixed	110	59	49	2
Ashkenazi Jewish	1,454	338	256	860
Latino and Indigenous people of the Americans	201	176	17	8
East Asian	1,312	1,276	34	2
European	11,864	5,470	3,158	3,236
South Asian	207	198	7	2
Central Asian	85	73	10	2
Middle Eastern	149	131	4	14
Finnish	31	20	5	6
Complex Admixture	71	58	8	5
**Total**	**15,742**	**7,957**	**3,643**	**4,142**

[1] Other phenotypes include atypical parkinsonism, e.g., progressive supranuclear palsy (PSP), multi system atrophy (MSA), corticobasal degeneration/syndrome (CBD/CBS), and dementia with Lewy bodies (DLB), as well as prodromal PD.

Supplementary Table 6: Study participant demographic information for NeuroBooster array genotyping data from GP2.

Ancestry	Total	PD	Controls	Other phenotypes [1]
African	2,643	942	1,679	22
African Admixed	1,111	285	801	25
Ashkenazi Jewish	2,655	1,292	411	952
Latino and Indigenous people of the Americans	646	458	155	33
East Asian	5,167	2,662	2,461	44
European	38,839	21,198	9,214	8,427
South Asian	643	391	226	26
Central Asian	903	552	343	8
Middle Eastern	581	311	225	45
Finnish	114	98	8	8
Complex Admixture	851	525	302	24
**Total**	**54,153**	**28,714**	**15,825**	**9,614**

[1] Other phenotypes include atypical parkinsonism, e.g., progressive supranuclear palsy (PSP), multi system atrophy (MSA), corticobasal degeneration/syndrome (CBD/CBS), and dementia with Lewy bodies (DLB), as well as prodromal PD.

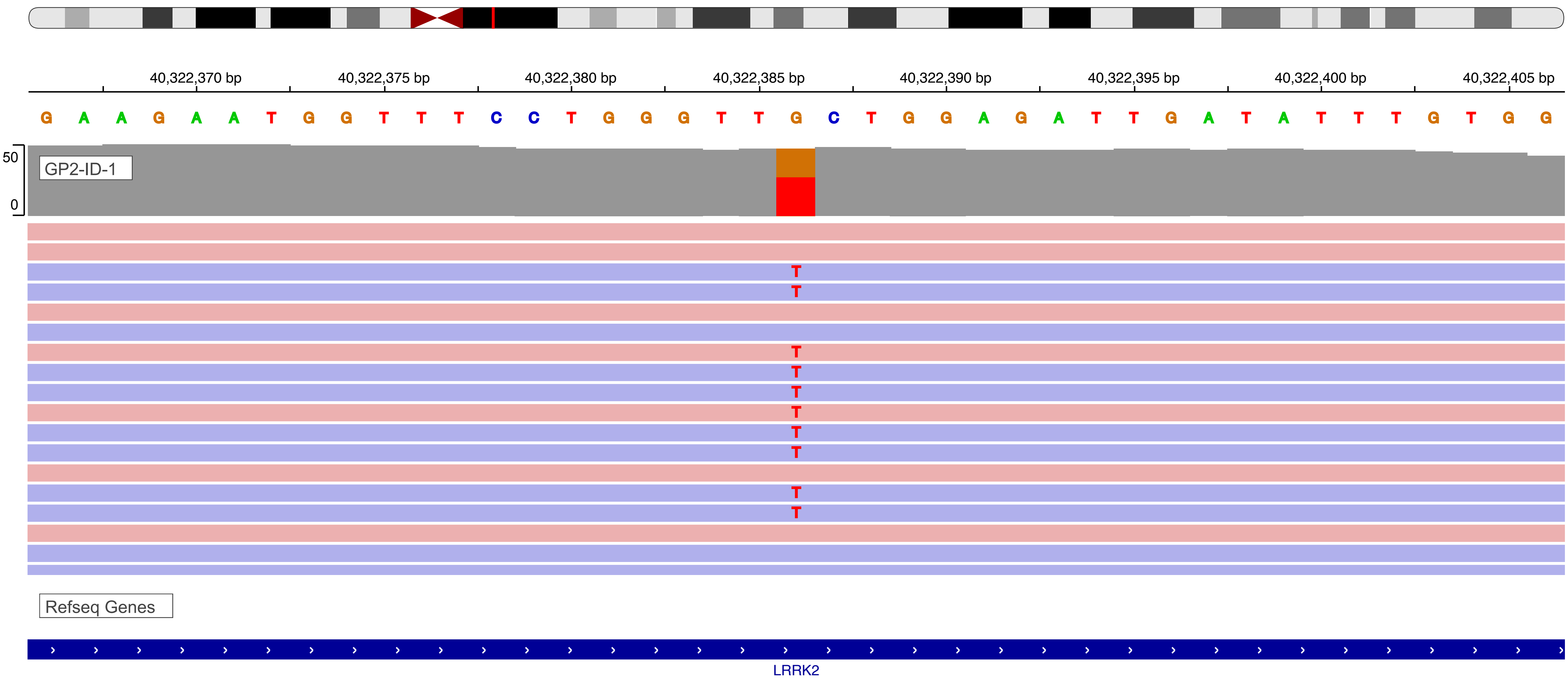

Supplementary Figure 1. Integrative Genomics Viewer window of *LRRK2* p.L1795F variant (chr12:40322386:G:T) identified in individual GP2-ID-1

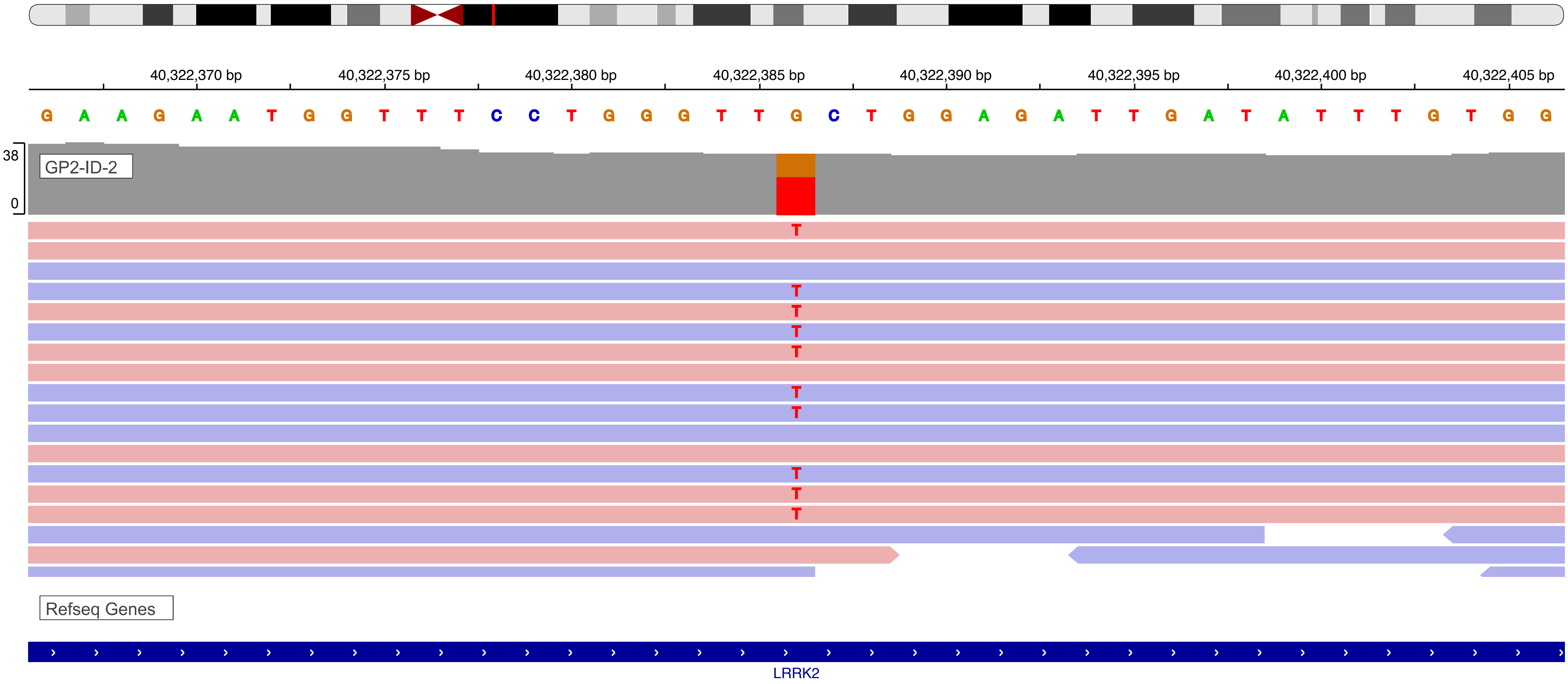

Supplementary Figure 2. Integrative Genomics Viewer window of *LRRK2* p.L1795F variant (chr12:40322386:G:T) identified in individual GP2-ID-2

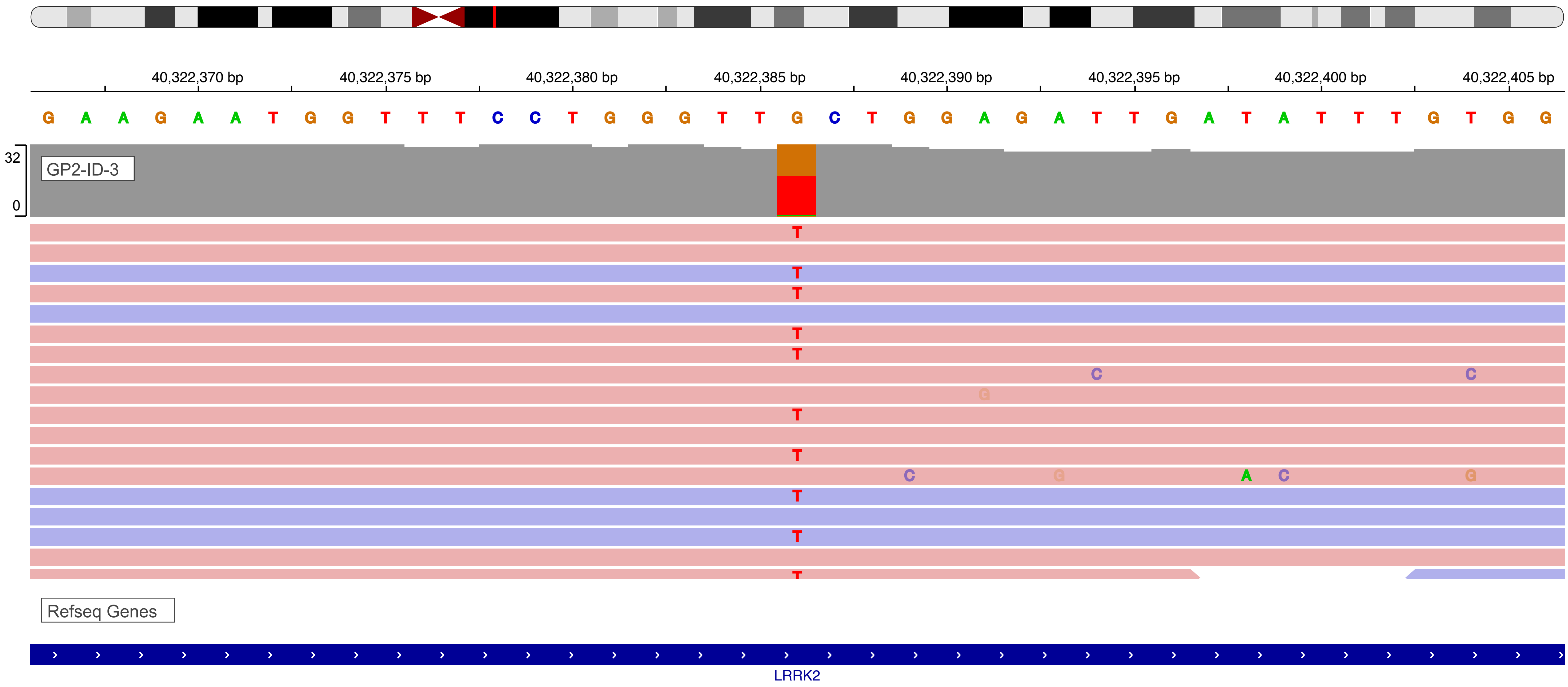

Supplementary Figure 3. Integrative Genomics Viewer window of *LRRK2* p.L1795F variant (chr12:40322386:G:T) identified in individual GP2-ID-3

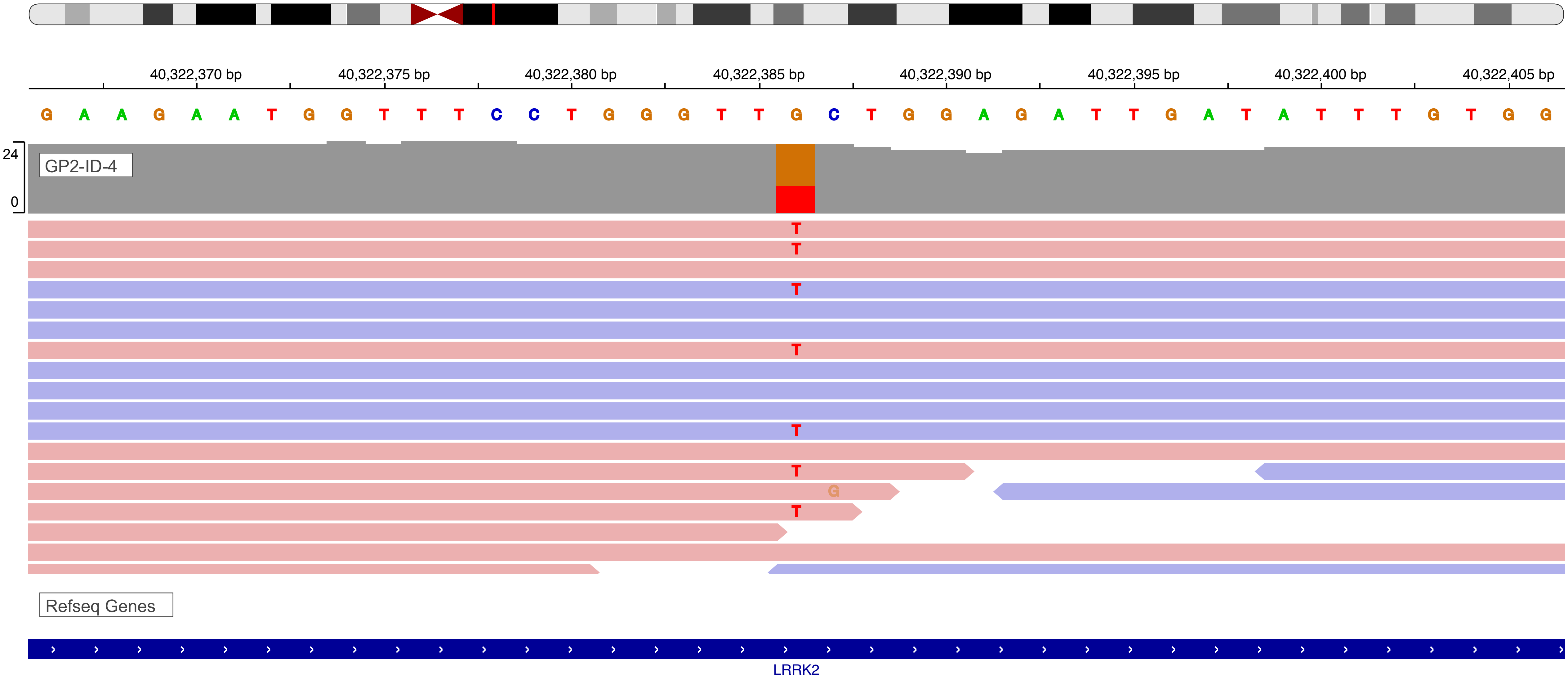

Supplementary Figure 4. Integrative Genomics Viewer window of *LRRK2* p.L1795F variant (chr12:40322386:G:T) identified in individual GP2-ID-4

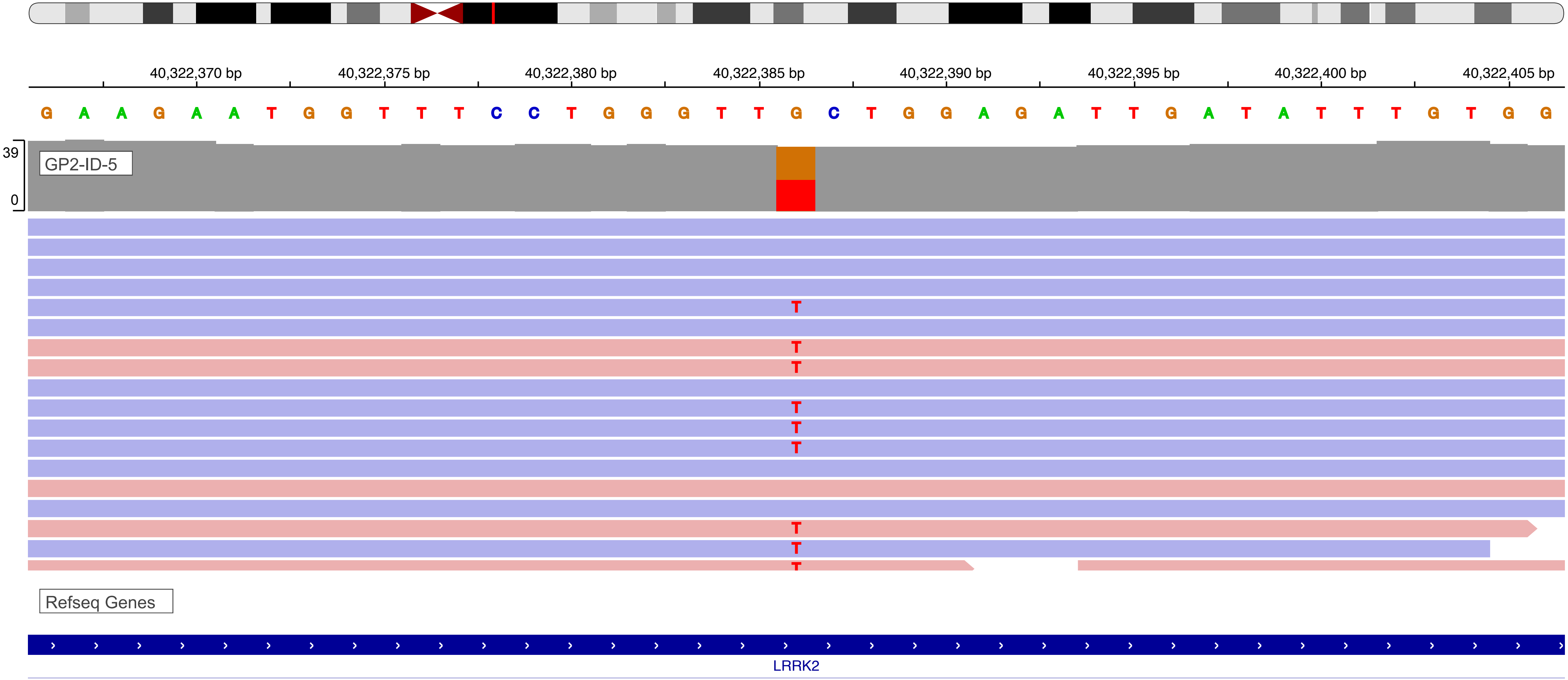

Supplementary Figure 5. Integrative Genomics Viewer window of *LRRK2* p.L1795F variant (chr12:40322386:G:T) identified in individual GP2-ID-5

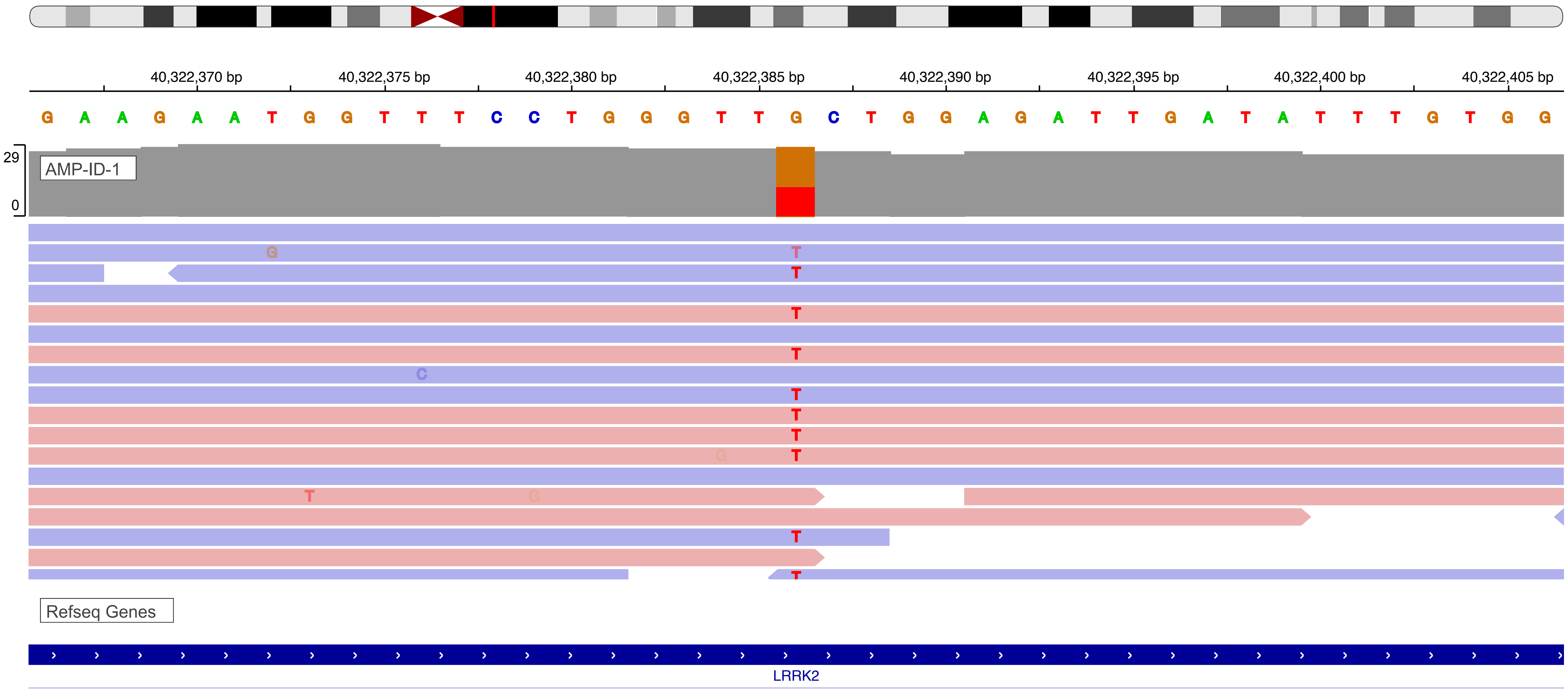

Supplementary Figure 6. Integrative Genomics Viewer window of *LRRK2* p.L1795F variant (chr12:40322386:G:T) identified in individual AMP-ID-1

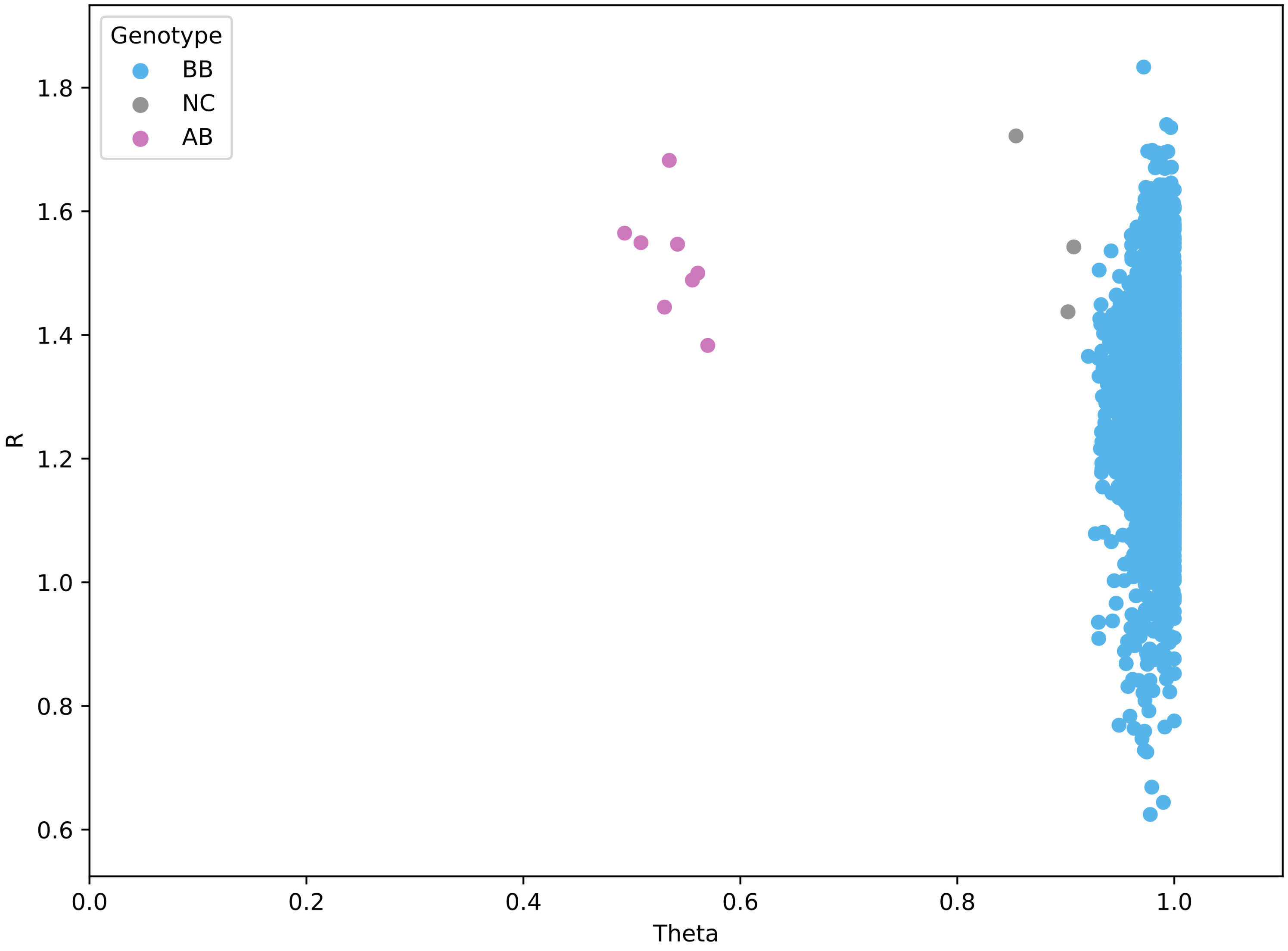

Supplementary Figure 7. Genotype intensity plot confirming the heterozygous genotype of the samples carrying *LRRK2* p.L1795F variant. Genotypes are called for each sample (dot) by their normalized signal intensity (R) and normalized Allele Frequency (Theta) for the probe genotyping *LRRK2* p.L1795F (grey = genotype not called, blue = BB, pink = AB, A= the mutant allele)

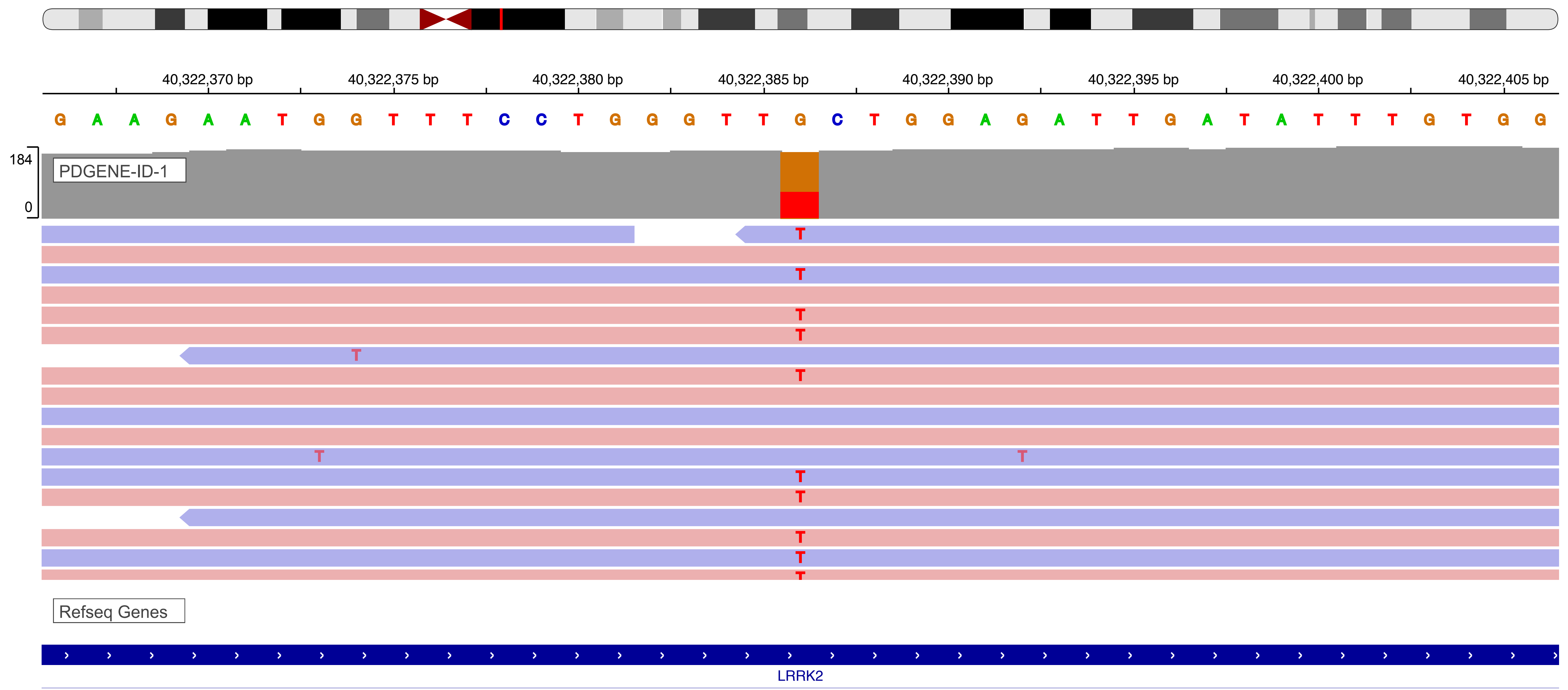

Supplementary Figure 8. Integrative Genomics Viewer window of *LRRK2* p.L1795F variant (chr12:40322386:G:T) identified in individual PDGENE-ID-1

Species	Match	AA	Alignment
Human		1795	IDSLMEEWFPGLLEIDICGEGETL
mutated		1795	IDSLMEEWFPG**F**LEIDI
Ptroglodytes	all identical	1795	IDSLMEEWFPGLLEIDI
Mmulatta	no homologue		
Fcatus	all identical	1795	EWFPGLLEIDICGEGET
Mmusculus	all identical	1795	IDSLMEEWFPGLLEI
Ggallus	all identical	1793	IDSLMEEWFPGLLDIDVCGEGET
Trubripes	no homologue		
Drerio	all identical	1791	EEWFPGLLTTDIHGTGET
Dmelanogaster	no alignment		
Celegans	not conserved	1567	LLEDWYPA**LG**TRFVHSSEG
Xtropicalis	all identical	1782	IDSLMEEWFPGLLESGICEEGEA

Supplementary Figure 9. Conservation of the amino acid (AA) Leucine at position 1795 across different species. Adapted from MutationTaster (https://www.mutationtaster.org).
